# Hepatoblastoma: A Need for Cell Lines and Tissue Banks to Develop Targeted Drug Therapies

**DOI:** 10.3389/fped.2016.00022

**Published:** 2016-03-21

**Authors:** Rishi Raj Rikhi, Kimberlee K. Spady, Ruth I. Hoffman, Michael S. Bateman, Max Bateman, Lisa Easom Howard

**Affiliations:** ^1^Children’s Cancer Therapy Development Institute, Beaverton, OR, USA; ^2^Faculty of the 2015 Pediatric Cancer Biology Nanocourse, Children’s Cancer Therapy Development Institute, Fort Collins, CO, USA

**Keywords:** hepatoblastoma, roadmap, xenograft models, cell lines, tissue procurement

## Abstract

Limited research exists regarding the most aggressive forms of hepatoblastoma. Cell lines of the rare subtypes of hepatoblastoma with poor prognosis are not only difficult to attain but also challenging to characterize histologically. A community-driven approach to educating parents and families, regarding the need for donated tissue, is necessary for scientists to have access to resources for murine models and drug discovery. Herein, we describe the currently available resources, existing gaps in research, and the path to move forward for uniform cure of hepatoblastoma.

## Introduction

Hepatoblastoma is the most common primary liver tumor diagnosed in childhood (1), with approximately 100 cases in the U.S. annually (2). Despite a high cure rate for those children whose tumor is resectable, there remains a group of children for whom a cure is out of reach.

The disease predominantly occurs in young children, from birth to 5 years of age (1). The histological subtypes of hepatoblastoma are fetal, embryonal, mixed epithelial–mesenchymal, and small cell undifferentiated (3). However, there is currently a lack of understanding regarding the origins and pathophysiology of these different subtypes of hepatoblastoma.

Clinically, the empirically driven advancements in postoperative chemotherapy and surgery, including the multidisciplinary approach set forth through the Pretreatment Extent of Disease guidelines (1), has improved outcomes for hepatoblastoma. These guidelines rely on a standardized staging system using imaging for detecting amount of tumor involvement (4). Despite these clinical advancements, the more aggressive forms of hepatoblastoma remain difficult to treat. Current treatments for aggressive forms of hepatoblastoma include doxorubicin, irinotecan (clinical trials), hepatic artery chemoembolization in addition to chemotherapy agents, as well as liver transplantation or partial resection with neoadjuvant chemotherapy (5).

Scientists and clinicians are now seeking non-chemotherapeutic treatments for patients with unresectable or metastatic tumor – treatments that directly target the molecular underpinnings of hepatoblastoma progression. For example, clinical trials regarding cixutumumab and pazopanib, monoclonal antibodies, and alisertib, a kinase inhibitor, have all been completed or are actively being investigated in phase 2 clinical trails for the treatment of refractory hepatoblastoma (6).

In order to find other targeted therapies, researchers need hepatoblastoma tissues and cell lines. There is an absence of diversity in hepatoblastoma cell lines for scientists and clinicians to use to better understand the disease. In this paper, we describe the need for more cell lines and murine models to advance the discovery of therapeutic targets for the more aggressive subtypes of hepatoblastoma.

## Methods

An extensive literature review of hepatoblastoma *via* PubMed was conducted to obtain information on research with unique hepatoblastoma cell lines and murine models. First, the authors found the number of distinct hepatoblastoma cell lines published in the literature. The search terms used were “‘hepatoblastoma’ [All Fields] and ‘cell line’ [MeSH Terms].” The search provided approximately 450 publications, all reviewed by the authors of this paper. Any publication that had a focus on hepatoblastoma was mentioned in Table 1. For each unique cell line, the authors attempted to find the primary article characterizing the cell line. Secondary publications using hepatoblastoma cell lines were also included in Table 1.

**Table 1:** **Literature review of hepatoblastoma cell lines and mouse models**.

Cell lines	Name/ID	Public availability	Age	Gender (m/f)	Year made	Histological subtype	Mutations	Primary reference	Secondary reference
**True hepatoblastoma**
	Hep G2	ATCC	15 years	m	1975	Epithelial	CTNNB1; Δ116 aa, 25–140, exon 3 and 4	(7, 8)	(9)
	HUH6	JCRB	12 months	m	1985	Mixed; predominant embryonal	CTNNB1; T41A	(10)	(9, 11)
	HepT1 (DZ25)	Dr. Steven Warmann (Germany)	34 months	f		Embryonal, poorly differentiated	CTNNB1; Δ76 aa, 5–80 exon 3	(12)	(9)
	HepT3 (tumor D204)		9 months	m		Fetal and embryonal	CTNNB1; T41A	(9)	(13)
	Hep293TT		5 years	f		Mixed; predominant embryonal	CTNNB1; Δ117aa	(14)	
	HepT8							(15)	
	HepT4							(15)	
	HepT5 (tumor D717)					Epithelial	CTNNB1; Δ76 amino acids, exon 3	(15)	
	HepT2 (tumor D166)		48 months	m		Epithelial	CTNNB1	(15)	
	HepU1		53 months	m		Fetal and embryonal		(16)	
	HepU2		58 months	m		Fetal and embryonal		(16)	
	OHR		4 months	m		Anaplastic (no alpha fetal protein)	TP53; R281H	(17)	(18)
	USM		11 months	f					(18)
	HB1		6 months	m		Mixed fetal and mesenchymal		(19)	
	c-HB3		1 years	m	1979	Well-differentiated fetal		(20)	
**Unsure hepatoblastoma**
	SMMC7721							(21)	
	Hep 3b							(8)	(22)
	HUH-7							(23)	(11)
	WRL-68							(24, 25)	
	COG-H-430 (unpublished)	COG						Unpublished	
**Hepatoblastoma-derived fibroblasts**
	GM08206 (unpublished)	Coriell Institute	7 months	m		Fibroblast		Unpublished	
**Mouse models**					**Metastatic (y/n)**		**Comments**		
**Chemically induced**
	B6C3F1; diethylnitrosamine (DEN) and sodium phenobarbital (PB)					Embryonal or small cell type	62% hepatoblastoma penetrance; HCA and HCC occurred in 54%	(26)	
**Transgenic**
	Cited1-CreERTM-GFP; Ctnnb1þ/ex3(fl)		8 weeks		y	Embryonal undifferentiated, pure fetal hepatoblastomas	38% hepatoblastoma penetrance; HCC occurred as well	(27)	
	ApoE-LIN28B		6 months			Fetal and cholangioblastic pattern	100 hepatoblastoma penetrance; HCC occurred as well	(28)	
	LAP-MYC					Mixed embryonal and fetal, predominant embryonal		(28)	
	Alb-MYC					Mixed embryonal and fetal, predominant embryonal		(28)	
**Cell line-derived xenografts**
**Subcutaneous**									
	Nu/nu Balb/c mice w/2 × 10^7^ HuH6 cells					Mixed; predominant embryonal		(29–31)	
	NMRI-Foxn1nu w/2–3 × 10^6^ HuH6 cells			f			Paravertebral areas	(32)	
	NOD/LtSz-scid IL2Rγnull 2 × 10^6^ HuH6 cells						Paravertebral areas	(33)	
	Athymic nude mice w/1 × 10^7^ HepG2 cells			f			Left flank	(34)	
	NOD/SCID immunodeficient w/5 × 10^5^ HuH6 cells			m				(35)	
	NOD/SCIDw/1 × 10^5^ HepG2 cells		14 days					(36)	
	Nude mice w/USM cells							(18)	
	Atyhmic nude mice w/2 × 10^6^ HepG2 cells		3 weeks	m			Left thigh	(37)	
	Nude mice (BALB/c, nu/nu) w/2 mm HB3 tissue cubes		14.5 days	f		Well differentiated fetal	Back	(20)	
	NOD/LtSz-scid IL2Rγnull mice w/2 × 10^6^ HUH6 cells		4 weeks				Paravertebral areas	(38)	
**Orthotopic**									
	NOD/LtSz-scid IL2Rγnull w/1 × 10^6^ HuH6 cells		5 weeks		n		83% hepatoblastoma penetrance – injected intrasplenically, no tumor growth *via* intravenous or intraperitoneal injection	(13)	
	NOD/LtSz-scid IL2Rγnull w/1 × 10^6^ HepT1 cells		5 weeks		n		50% hepatoblastoma penetrance – injected intrasplenically, no tumor growth *via* intravenous or intraperitoneal injection	(13)	
	NOD.Cg-Prkdcscid-IL2rgtmWjl/Sz w/1 × 10^6^ HuH6 cells		4 weeks		y	Embryonal	82% penetrance – injected intrasplenically	(39)	
**Patient-derived xenografts**
	HB-213	XenTech	19 months	f	y			(40)	
	HB-214	XenTech	30 months	f	y	Small cell undifferentiated		(40)	
	HB-217	XenTech	24 months	m	n			(40)	
	HB-229	XenTech	54 months	m	y			(40)	
	HB-232	XenTech	6 months	m	n			(40)	
	HB-233	XenTech	16 months	m	n	Small cell undifferentiated		(40)	
	HB-236	XenTech	8 months	f	n			(40)	
	HB-238	XenTech	110 months	f	n			(40)	
	HB-239	XenTech	113 months	m	n	Small cell undifferentiated		(40)	
	HB-243	XenTech	52 months	m	n			(40)	
	HB-244	XenTech	114 months	m	n			(40)	
	HB-252	XenTech	14 months	f	n			(40)	

Manuscripts that had differing characterizations of certain cell lines were included in the “Unsure Hepatoblastoma” portion of Table 1.

Next, the authors reviewed literature *via* PubMed to find murine models of hepatoblastoma. The search terms used were “hepatoblastoma murine models.” This provided approximately 50 publications that the authors reviewed. All publications that studied murine models of hepatoblastoma were included in Table 1. The models were classified as chemically induced, transgenic, and cell-derived xenografts. Furthermore, the xenograft studies were sorted based on subcutaneous or orthotopic models.

Finally, the authors searched for patient-derived xenografts *via* PubMed and found no manuscript publications. The authors then searched the *European Journal of Cancer* using the term “hepatoblastoma xenograft” and found a published abstract using patient-derived xenografts, which is included in Table 1.

## Results

Data regarding histological characterization and experimental murine models from only a few hepatoblastoma cell lines exist. These cell lines tend to have a favorable histology, leading to an underrepresentation of the high-risk subtypes (41).

### Cell Lines

Fifteen hepatoblastoma cell lines are described in current literature (Table 1). Additionally, there are four cell lines that are potentially hepatoblastoma, but significant inconsistencies in the literature render the data obtained from these lines unreliable. Even among the confirmed hepatoblastoma cell lines, however, there are many documented instances in which cell lines were mistaken for hepatocellular carcinoma (7). A fibroblast cell line harvested from the liver of a Beckwith–Wiedemann syndrome patient with hepatoblastoma is described (Table 1).

Most confirmed cell lines are of the mixed histology subtype. However, there are no cell lines of the small cell undifferentiated subtype, which carries the worst prognosis (7).

### Murine Models

Only one chemically induced murine model of hepatoblastoma has been reported (Table 1). Although four different transgenic murine models are described in the literature, these murine models were not specifically developed for the purpose of modeling hepatoblastoma. The transgenic murine models phenotypically express both hepatocellular carcinoma and hepatoblastoma (Table 1).

Ten unique cell line-derived subcutaneous xenografts and three cell line-derived orthotopic murine models of human hepatoblastoma exist (Table 1). These models primarily utilize the Hep G2 and HuH6 cells lines. Twelve unique patient-derived xenografts exist (Table 1).

### Potential Genetic Targets for Aggressive Hepatoblastoma

Many studies have noted genetic mutations specific to histological subtypes of hepatoblastoma (42). Hepatoblastoma cells have shown gain of 2q, 1q, Xp, and Xq; loss of 4q, 2q, and 1q; and loss of heterozygosity of insulin growth factor 2 (5). Subtypes with increased Notch expression are of the fetal subtype and tend to have a better prognosis. Those with overexpression of the Wingless-type MMTV Integration Site Family pathway are of the small cell undifferentiated subtype and carry a less favorable prognosis (3). Additionally, the more aggressive forms of hepatoblastoma have telomerase reverse transcriptase promoter mutations (43). Blocking the Wingless-type MMTV Integration Site Family pathway using NK1R antagonists has been shown to slow the progression of hepatoblastoma cell growth *in vitro* (44). Hepatoblastoma cells show an increase in activity of the hedgehog pathway, and abnormal signaling has been linked to more malignant potential (45). Forkhead Box G1 is overexpressed in hepatoblastoma, specifically the more aggressive subtypes, when compared to the fetal subtype (46).

## Discussion

In order to find targeted therapeutic options for hepatoblastoma, basic science studies need to be conducted. The few cell lines characterized and the inconsistencies in the literature on certain cell lines provide a major hurdle toward this goal. In addition, the availably of the cell lines is limited, which explains the narrow spectrum of cell lines used to derive xenografts from the already few hepatoblastoma cell lines. Additionally, diversity of histological subtypes is needed in order to find better treatment modalities for the more aggressive forms of hepatoblastoma. Interestingly, expression of fibroblasts enhances the growth of hepatoblastoma (47), which is why the hepatoblastoma-derived fibroblast cell line, GM08206 (Table 1), carries potential for more advanced studies. It is of important note that the majority of liver cells are aneuploid, which has been thought to protect the liver from chronic injury (48). Culturing surrounding normal liver tissue in addition to the tumor would provide insight into premalignant tissue field effect at the site of the tumor (46).

Certain repositories for hepatoblastoma are in the early stages of developing around the world, providing optimism for advancements in basic science research, and potentially leading to clinical trials for hepatoblastoma. The Children’s Oncology Group developed a Rare Tumor Committee that has lead to promising clinical trials for rare pediatric cancer (49), including a current clinical trial involving combination chemotherapy for different stages of hepatoblastoma. Although histological analysis is not used in the staging process, this trial presents the opportunity to provide awareness of hepatoblastoma and an opportunity to increase tissue donation. Currently, the Children’s Oncology group has one hepatoblastoma cell line (COG-H-430), not available on the open distribution list, but can be obtained with a materials transfer agreement (personal communication).

In addition to the Children’s Oncology Group, the Japanese Collection of Research Biosources hosts a cell bank that provided the cell lines for the majority of hepatoblastoma manuscripts in the literature review (9). However, currently only HUH6 is available for public distribution. Many published hepatoblastoma cell lines found in the literature review were not within the last decade, which could explain the difficulty in obtaining certain cell lines today.

Most importantly, many international groups, such as Childhood Liver Tumors Strategy Group and the Society for Pediatric Oncology and Hematology, have collaborated with Children’s Oncology Group and the Japanese Collection of Research Biosources, which initially led to the Pretreatment Extent of Disease guidelines (50). It is this type of collaboration that can result in an increase in cell lines and tissue-banking repositories. One example is the Childhood Liver Tumors Strategy Group, which runs a tissue bank for childhood liver tumors (51).

Recently, further collaboration has allowed for the Children’s Hepatic tumors International Collaboration, to obtain data on 1,605 hepatoblastoma patients, aimed at creating a database to identify prognostic factors for this rare pediatric cancer (52). One limitation to the database, mentioned by the authors, was the exclusion of histology due to the lack of international consensus in characterizing subtypes (52).

As more interaction among family members is made, newer registries, in addition to those previously mentioned, will continue to grow. The Macy Easom Foundation has committed to funding development of the Hepatoblastoma Registry, as well as the expense of administration, data compilation, and analysis (18).

Despite the current development of repositories, an increase in cell lines and murine models available for research purposes cannot progress unless methods are in place to increase awareness for tissue donation in hepatoblastoma. Both parents and treating physicians must be made aware of the need for hepatoblastoma tissue and the opportunity to support research *via* autopsy tissue donations. The decision, whether to make an autopsy tissue donation, is difficult, intensely personal, and unique for each family. The authors recognize the delicate balance between making parents aware of the need and opportunity while taking care to respect every family’s response and perspective.

A parent who wishes to arrange for an autopsy donation should not be burdened with making the arrangements. With parents’ consent, volunteers and professionals must be in place to make the necessary contacts and establish logistics of the donation. These arrangements may include contacting the treating physician, speaking with the local pathologist who will perform the autopsy, connecting the researcher who will receive the donated tissue with the pathologist, and arranging for transport of the body from the child’s home to the hospital (and return to the funeral home).

Grassroots communication and interaction among family members, caregivers, and others affected by a particular diagnosis has significantly influenced progress in some areas of pediatric cancer research. As an example, interaction among families affected by diffuse intrinsic pontine glioma (DIPG) in an online discussion group is considered by some to be the first step in raising a tide turning awareness in that community. The result was a promising therapeutic drug, panobinostat, for treatment (53).

**Figure 1:**
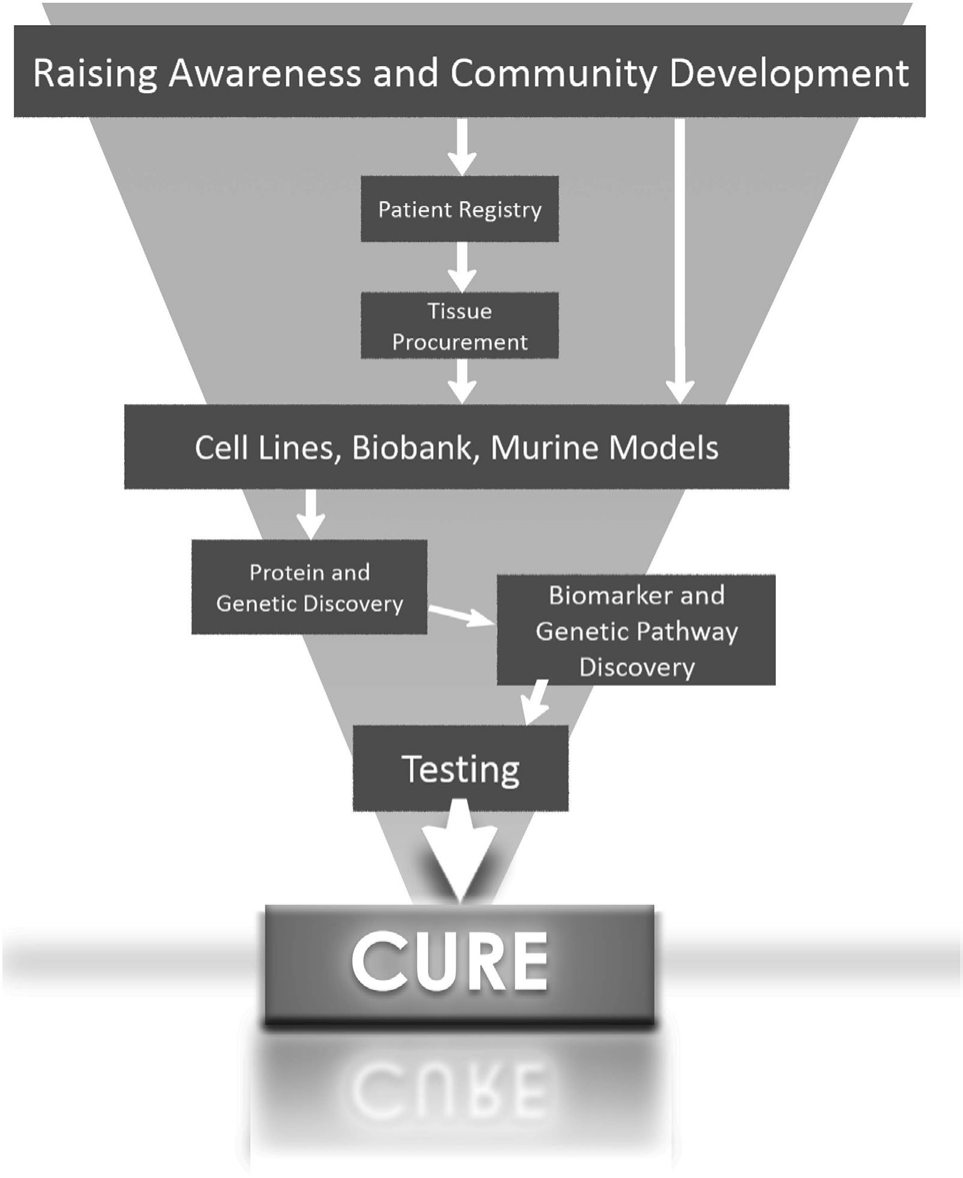
**Roadmap to finding a cure for hepatoblastoma**.

In summary, the greatest potential for the development of targeted therapy for aggressive forms of hepatoblastoma will come when scientists have access to hepatoblastoma cells lines and tissues with histological subtype diversity (Figure 1).

## Author Contributions

RR, KS, RH, MSB, MB, and LH: contributed significantly to the acquisition, intellectual content, and final approval and are in agreement with all aspects of the work.

## Conflict of Interest Statement

The authors declare that the research was conducted in the absence of any commercial or financial relationships that could be construed as a potential conflict of interest.

## References

[1] Khaderi S Guiteau J Cotton RT O’Mahony C Rana A Goss JA. Role of liver transplantation in the management of hepatoblastoma in the pediatric population. World J Transplant (2014) 4(4):294–8. 10.5500/wjt.v4.i4.294 25540737 PMC4274598

[2] Johnson KJ Williams KS Ross JA Krailo MD Tomlinson GE Malogolowkin MH Parental tobacco and alcohol use and risk of hepatoblastoma in offspring: a report from the children’s oncology group. Cancer Epidemiol Biomarkers Prev (2013) 22(10):1837–43. 10.1158/1055-9965.EPI-13-0432 23950215 PMC4188411

[3] Cairo S Armengol C De Reynies A Wei Y Thomas E Renard CA Hepatic stem-like phenotype and interplay of Wnt/beta-catenin and Myc signaling in aggressive childhood liver cancer. Cancer Cell (2008) 14(6):471–84. 10.1016/j.ccr.2008.11.002 19061838

[4] Pappo AS Furman WL Schultz KA Ferrari A Helman L Krailo MD. Rare tumors in children: progress through collaboration. J Clin Oncol (2015) 33(27):3047–54. 10.1200/JCO.2014.59.3632 26304909 PMC4979197

[5] Venkatramani R Furman WL Fuchs J Warmann SW Malogolowkin MH. Current and future management strategies for relapsed or progressive hepatoblastoma. Paediatr Drugs (2012) 14(4):221–32. 10.2165/11597740-000000000-00000 22702740

[6] Cixutumumab in Treating Patients with Relapsed or Refractory Solid Tumors clinicaltrials.gov (2015). Available from: https://clinicaltrials.gov/ct2/show/study/NCT00831844?term=hepatoblastoma&rank=9&sect=X70156

[7] Lopez-Terrada D Cheung SW Finegold MJ Knowles BB Hep G2 is a hepatoblastoma-derived cell line. Hum Pathol (2009) 40(10):1512–5. 10.1016/j.humpath.2009.07.003 19751877

[8] Zannis VI Breslow JL SanGiacomo TR Aden DP Knowles BB. Characterization of the major apolipoproteins secreted by two human hepatoma cell lines. Biochemistry (1981) 20(25):7089–96. 10.1021/bi00528a006 6274388

[9] Koch A Denkhaus D Albrecht S Leuschner I von Schweinitz D Pietsch T. Childhood hepatoblastomas frequently carry a mutated degradation targeting box of the beta-catenin gene. Cancer Res (1999) 59(2):269–73. 9927029

[10] Tokiwa T Doi I Sato J. Preparation of single cell suspensions from hepatoma cells in culture. Acta Med Okayama (1975) 29(2):147–50. 169672

[11] Purcell R Childs M Maibach R Miles C Turner C Zimmermann A HGF/c-Met related activation of beta-catenin in hepatoblastoma. J Exp Clin Cancer Res (2011) 30:96 10.1186/1756-9966-30-96 21992464 PMC3207961

[12] Pietsch T Fonatsch C Albrecht S Maschek H Wolf HK von Schweinitz D. Characterization of the continuous cell line HepT1 derived from a human hepatoblastoma. Lab Invest (1996) 74(4):809–18. 8606490

[13] Ellerkamp V Armeanu-Ebinger S Wenz J Warmann SW Schafer J Ruck P Successful establishment of an orthotopic hepatoblastoma in vivo model in NOD/LtSz-scid IL2Rgammanull mice. PLoS One (2011) 6(8):e23419 10.1371/journal.pone.0023419 21853130 PMC3154467

[14] Chen TT Rakheja D Hung JY Hornsby PJ Tabaczewski P Malogolowkin M Establishment and characterization of a cancer cell line derived from an aggressive childhood liver tumor. Pediatr Blood Cancer (2009) 53(6):1040–7. 10.1002/pbc.22187 19637320

[15] Koch A Waha A Hartmann W Hrychyk A Schuller U Waha A Elevated expression of Wnt antagonists is a common event in hepatoblastomas. Clin Cancer Res (2005) 11(12):4295–304. 10.1158/1078-0432.CCR-04-1162 15958610

[16] Scheil S Hagen S Bruderlein S Leuschner I Behnisch W Moller P. Two novel in vitro human hepatoblastoma models, HepU1 and HepU2, are highly characteristic of fetal-embryonal differentiation in hepatoblastoma. Int J Cancer (2003) 105(3):347–52. 10.1002/ijc.11082 12704668

[17] Kanno S Tsunoda Y Shibusawa K Ishikawa M Okamoto S Matsumura M [Establishment of a human hepatoblastoma (immature type) cell line OHR]. Hum Cell (1989) 2(2):211. 2562091

[18] Ohnishi H Kawamura M Hanada R Kaneko Y Tsunoda Y Hongo T Infrequent mutations of the TP53 gene and no amplification of the MDM2 gene in hepatoblastomas. Genes Chromosomes Cancer (1996) 15(3):187–90. 10.1002/(SICI)1098-2264(199603)15:3<187::AID-GCC8>3.0.CO;2-Z 8721685

[19] Manchester KM Warren DJ Erlandson RA Wheatley JM La Quaglia MP. Establishment and characterization of a novel hepatoblastoma-derived cell line. J Pediatr Surg (1995) 30(4):553–8. 10.1016/0022-3468(95)90129-9 7595832

[20] Hata Y Uchino J Sato K Sasaki F Une Y Naito H Establishment of an experimental model of human hepatoblastoma. Cancer (1982) 50(1):97–101. 10.1002/1097-0142(19820701)50:1<97::AID-CNCR2820500118>3.0.CO;2-4 6177394

[21] Feng X Tang Z Zheng Z. [Preliminary studies on the effects of tumor necrosis factor gene transfer on the growth of human hepatocellular carcinoma cells in nude mice]. Zhonghua Zhong Liu Za Zhi (1995) 17(3):167–9. 7656817

[22] Delgado ER Yang J So J Leimgruber S Kahn M Ishitani T Identification and characterization of a novel small-molecule inhibitor of beta-catenin signaling. Am J Pathol (2014) 184(7):2111–22. 10.1016/j.ajpath.2014.04.002 24819961 PMC4076560

[23] Nakabayashi H Taketa K Miyano K Yamane T Sato J. Growth of human hepatoma cells lines with differentiated functions in chemically defined medium. Cancer Res (1982) 42(9):3858–63. 6286115

[24] Hsu IC Tokiwa T Bennett W Metcalf RA Welsh JA Sun T p53 gene mutation and integrated hepatitis B viral DNA sequences in human liver cancer cell lines. Carcinogenesis (1993) 14(5):987–92. 10.1093/carcin/14.5.987 8389256

[25] Falk PM Sabater RT Carballo DD Jr. Response of the human hepatic tissue cultures Hep-G2 and WRL-68 to cocaine. J Pharmacol Toxicol Methods (1995) 33(2):113–20. 10.1016/1056-8719(94)00065-C 7766918

[26] Sakairi T Kobayashi K Goto K Okada M Kusakabe M Tsuchiya T Greater expression of transforming growth factor alpha and proliferating cell nuclear antigen staining in mouse hepatoblastomas than hepatocellular carcinomas induced by a diethylnitrosamine-sodium phenobarbital regimen. Toxicol Pathol (2001) 29(4):479–82. 10.1080/01926230152499962 11560253

[27] Mokkapati S Niopek K Huang L Cunniff KJ Ruteshouser EC deCaestecker M Beta-catenin activation in a novel liver progenitor cell type is sufficient to cause hepatocellular carcinoma and hepatoblastoma. Cancer Res (2014) 74(16):4515–25. 10.1158/0008-5472.CAN-13-3275 24848510 PMC4134699

[28] Nguyen LH Robinton DA Seligson MT Wu L Li L Rakheja D Lin28b is sufficient to drive liver cancer and necessary for its maintenance in murine models. Cancer Cell (2014) 26(2):248–61. 10.1016/j.ccr.2014.06.018 25117712 PMC4145706

[29] Berger M Neth O Ilmer M Garnier A Salinas-Martin MV de Agustin Asencio JC Hepatoblastoma cells express truncated neurokinin-1 receptor and can be growth inhibited by aprepitant in vitro and in vivo. J Hepatol (2014) 60(5):985–94. 10.1016/j.jhep.2013.12.024 24412605

[30] Wagner F Henningsen B Lederer C Eichenmuller M Godeke J Muller-Hocker J Rapamycin blocks hepatoblastoma growth in vitro and in vivo implicating new treatment options in high-risk patients. Eur J Cancer (2012) 48(15):2442–50. 10.1016/j.ejca.2011.12.032 22285179

[31] Cairo S Wang Y de Reynies A Duroure K Dahan J Redon MJ Stem cell-like micro-RNA signature driven by Myc in aggressive liver cancer. Proc Natl Acad Sci U S A (2010) 107(47):20471–6. 10.1073/pnas.1009009107 21059911 PMC2996672

[32] Eicher C Dewerth A Thomale J Ellerkamp V Hildenbrand S Warmann SW Effect of sorafenib combined with cytostatic agents on hepatoblastoma cell lines and xenografts. Br J Cancer (2013) 108(2):334–41. 10.1038/bjc.2012.539 23257893 PMC3566826

[33] Lieber J Eicher C Wenz J Kirchner B Warmann SW Fuchs J The BH3 mimetic ABT-737 increases treatment efficiency of paclitaxel against hepatoblastoma. BMC Cancer (2011) 11:362. 10.1186/1471-2407-11-362 21854558 PMC3176244

[34] Marotta DE Cao W Wileyto EP Li H Corbin I Rickter E Evaluation of bacteriochlorophyll-reconstituted low-density lipoprotein nanoparticles for photodynamic therapy efficacy in vivo. Nanomedicine (Lond) (2011) 6(3):475–87. 10.2217/nnm.11.8 21542686 PMC3137792

[35] Hayashi S Fujita K Matsumoto S Akita M Satomi A Isolation and identification of cancer stem cells from a side population of a human hepatoblastoma cell line, HuH-6 clone-5. Pediatr Surg Int (2011) 27(1):9–16. 10.1007/s00383-010-2719-x 20936478

[36] Mezzanotte L Fazzina R Michelini E Tonelli R Pession A Branchini B In vivo bioluminescence imaging of murine xenograft cancer models with a red-shifted thermostable luciferase. Mol Imaging Biol (2010) 12(4):406–14. 10.1007/s11307-009-0291-3 19937390

[37] Klein JL Nguyen TH Laroque P Kopher KA Williams JR Wessels BW Yttrium-90 and iodine-131 radioimmunoglobulin therapy of an experimental human hepatoma. Cancer Res (1989) 49(22):6383–9. 2553255

[38] Lieber J Dewerth A Wenz J Kirchner B Eicher C Warmann SW Increased efficacy of CDDP in a xenograft model of hepatoblastoma using the apoptosis sensitizer ABT-737. Oncol Rep (2013) 29(2):646–52. 10.3892/or.2012.2150 23229825

[39] Lieber J Ellerkamp V Vogt F Wenz J Warmann SW Fuchs J BH3-mimetic drugs prevent tumour onset in an orthotopic mouse model of hepatoblastoma. Exp Cell Res (2014) 322(1):217–25. 10.1016/j.yexcr.2013.12.007 24355809

[40] Fabre M Nicolle D Gorse A Déas O Mussini C Brugières L A panel of pediatric liver cancer patient-derived xenografts to improve stratification of children with hepatoblastoma. In: Pieters R, editor. European Journal of Cancer. ENA 2014: EORTC-NCI-AACR Symposium; 2014; Badalona, Spain. Oxford: Elsevier (2014). 25 p.

[41] Zhang Y Zhang WL Huang DS Hong L Wang YZ Zhu X Clinical effectiveness of multimodality treatment on advanced pediatric hepatoblastoma. Eur Rev Med Pharmacol Sci (2014) 18(7):1018–26. 24763882

[42] Lopez-Terrada D Gunaratne PH Adesina AM Pulliam J Hoang DM Nguyen Y Histologic subtypes of hepatoblastoma are characterized by differential canonical Wnt and Notch pathway activation in DLK+ precursors. Hum Pathol (2009) 40(6):783–94. 10.1016/j.humpath.2008.07.022 19200579

[43] Eichenmuller M Trippel F Kreuder M Beck A Schwarzmayr T Haberle B The genomic landscape of hepatoblastoma and their progenies with HCC-like features. J Hepatol (2014) 61(6):1312–20. 10.1016/j.jhep.2014.08.009 25135868

[44] Ilmer M Garnier A Vykoukal J Alt E von Schweinitz D Kappler R Targeting the neurokinin-1 receptor compromises canonical Wnt signaling in hepatoblastoma. Mol Cancer Ther (2015) 14(12):2712–21. 10.1158/1535-7163.MCT-15-0206 26516161

[45] Li YC Deng YH Guo ZH Zhang MM Zhu J Pu CL Prognostic value of hedgehog signal component expressions in hepatoblastoma patients. Eur J Med Res (2010) 15(11):468–74. 21159571 10.1186/2047-783X-15-11-468 PMC3352655

[46] Chai H Brown RE. Field effect in cancer-an update. Ann Clin Lab Sci (2009) 39(4):331–7. 19880759

[47] Asada N Tanaka Y Hayashido Y Toratani S Kan M Kitamoto M Expression of fibroblast growth factor receptor genes in human hepatoma-derived cell lines. In Vitro Cell Dev Biol Anim (2003) 39(7):321–8. 10.1290/1543-706X(2003)039<0321:EOFGFR>2.0.CO;2 14753849

[48] Duncan AW Hanlon Newell AE Bi W Finegold MJ Olson SB Beaudet AL Aneuploidy as a mechanism for stress-induced liver adaptation. J Clin Invest (2012) 122(9):3307–15. 10.1172/JCI64026 22863619 PMC3428097

[49] Kotecha RS Kees UR Cole CH Gottardo NG Rare childhood cancers – an increasing entity requiring the need for global consensus and collaboration. Cancer Med (2015) 4(6):819–24. 10.1002/cam4.426 25664881 PMC4472204

[50] Aronson DC Czauderna P Maibach R Perilongo G Morland B. The treatment of hepatoblastoma: its evolution and the current status as per the SIOPEL trials. J Indian Assoc Pediatr Surg (2014) 19(4):201–7. 10.4103/0971-9261.142001 25336801 PMC4204244

[51] Grotzer M History SIOPEL (2016). Available from: http://www.siopel.org/?q=node/47

[52] Czauderna P Haeberle B Hiyama E Rangaswami A Krailo M Maibach R The Children’s Hepatic tumors International Collaboration (CHIC): novel global rare tumor database yields new prognostic factors in hepatoblastoma and becomes a research model. Eur J Cancer (2016) 52:92–101. 10.1016/j.ejca.2015.09.023 26655560 PMC5141607

[53] Grasso CS Tang Y Truffaux N Berlow NE Liu L Debily MA Functionally defined therapeutic targets in diffuse intrinsic pontine glioma. Nat Med (2015) 21(6):555–9. 10.1038/nm.3855 25939062 PMC4862411

